# The prevalence of severe maternal morbidity and near miss and associated factors in Sergipe, Northeast Brazil

**DOI:** 10.1186/1471-2393-14-25

**Published:** 2014-01-16

**Authors:** Larissa Paes Leme Galvão, Fabiano Alvim-Pereira, Caio Menezes Machado de Mendonça, Filipe Emanuel Fonseca Menezes, Kaique Andre do Nascimento Góis, Ruy Farias Ribeiro Jr, Ricardo Queiroz Gurgel

**Affiliations:** 1 Federal University of Sergipe, Av. Beira Mar, 2016 ap. 402 Bairro 13 de julho, Aracaju, SE 49025-040, Brazil

**Keywords:** Severe maternal morbidity, Maternal deaths, Severe acute obstetric morbidity, Deliveries, Epidemiology, Maternal near miss

## Abstract

**Background:**

The investigation of severe maternal morbidity (SAMM) and maternal near miss (NM) and associated risk factors is important for the global reduction of maternal mortality. This study investigated the prevalence of SAMM and NM cases and the associated risk factors in two reference maternity hospitals in a capital city in Northeast-Brazil.

**Methods:**

A cross-sectional study with a nested case–control component was conducted from June-2011 to May-2012. Case identification was prospective and data collection was performed according to WHO criteria and definitions. *Odds ratio* with confidence intervals and multivariate analysis were used whenever possible.

**Results:**

There were 16,243 deliveries, 1,102 SAMM cases, 77 NM cases and 17 maternal deaths. The maternal NM outcome ratio was 5.8 cases/1,000 live births (LB); the total prevalence of SAMM + NM was 72.6 cases/1,000 LB, the maternal near miss: mortality ratio was 4.5cases/1 maternal death (18% of mortality index). Management-based criteria were the most common events for NM (87.1%) and hypertensive disorders for SAMM (67.5%). Higher age, previous abortion and caesarean delivery, the non-adhesion to antenatal care, current caesarean delivery and bad perinatal results were associated with SAMM/NM. In the multivariate analysis, patient’s status, previous caesarian and abortion and level of consciousness were significant when analyzed together.

**Conclusions:**

SAMM and NM situations were prevalent in the studied population and some risk factors seem to be associated with the event, particularly previous gestational antecedents. Protocols based on SAMM/NM situations can save lives and decrease maternal mortality.

## Background

Worldwide, approximately 800 women die every day from preventable causes related to pregnancy and delivery [1]. An additional ten million experience some kind of complication and a further three million neonatal deaths occur every year globally [2,3]. Most of these situations are preventable and 99% of maternal deaths occur in developing countries [1]. The maternal mortality ratio in Brazil is about 60.1/100,000 live births (LB) and a total of 1,719 deaths were estimated to have occurred in 2010. The main causes of maternal deaths this year were: hypertension (21.1%), hemorrhage (11.9%), complications of labor (9.6%), sepsis (8.6%), abortion (6.5%) and HIV and infectious disease (5%) [4]. Maternal deaths occur asymmetrically and vary according to the region in country and its state of development.

In most places, the study of maternal mortality has become increasingly difficult for several reasons, especially due to its rare occurrence. It is therefore very difficult to do population studies that aim to understand why these women die nowadays [5].

In 2009, the WHO established a set of criteria for SAMM and for NM in order to standardize data and calculate indicators for comparing different settings and identify cases of interest [6]. Severe acute maternal morbidity (SAMM) and maternal near miss (NM) are events involved in the biological *continuum* that goes from the normal expected healthy situation of a pregnancy to maternal death [7]. NM events are used to study situations where women survived, but nearly died due to a complication during pregnancy, delivery and postpartum and help to reduce maternal mortality by improving maternal health [8,9]. Despite these cases have several aspects in common with women who died (especially the same risk factors), fortunately these events are at least three times more frequent than maternal deaths. SAMM is a condition more broadly defined; the cases are less serious and represent a situation that precedes the NM situations in severity (Table four, [6]). NM situations are classified according to Table three in Say, Souza and Pattinson paper [6], are more specific and often represent the stage that immediately precedes maternal death [6].

A systematic review, including 82 studies from 46 countries, demonstrated that the prevalence ranged from 0.04% to 14.98% [10]. Other similar studies found NM incidence ratios of 3.4 cases/1,000 deliveries and 18 cases/1,000 deliveries [11]. A recent Brazilian multicenter study, which adopted the new SAMM and NM classification from the WHO (2009), analyzed 27 reference maternity hospitals in one year and found 116.3 cases/1,000LB [12].

Brazil is a country with large territorial and economic disparities, making it difficult to accurately estimate the current SAMM/NM situation. The majority of the studies are based on populations from large urban areas and this is not representative of the general population. What happens outside of these centers, especially in the poorest areas, also contributes to the national situation. This study aims to determine the prevalence of SAMM and NM events, to describe the associated risk factors and calculate the indicators recommended by the WHO in the two tertiary public services located in Aracaju, State Capital of Sergipe, in Northeast Brazil.

## Methods

A cross-sectional study with a nested case–control component was conducted to identify pregnant women who were at risk of SAMM and NM in the two reference maternity hospitals of Sergipe state, Northeast-Brazil, between June 2011 and May 2012. Case identification was prospective and data collection was performed concomitantly. The population studied is of a low socioeconomic level and the vast majority depend on the public health system. The two maternity hospitals used are the only reference maternity hospitals for the entire state: Santa Izabel Hospital and Nossa Senhora de Lourdes Maternity. The first institution is responsible for 800 deliveries per month, is the reference maternity hospital for low and medium obstetric risk and has the only intensive care unit for obstetric patients in the state. The second is the high risk maternity hospital for the state (350 deliveries per month), and hosts reference services and specialists from other clinical and surgical areas related to obstetric conditions. Both institutions have teams of obstetricians, anesthesiologists and neonatologists available all the time and are equipped with a blood bank, surgical theaters and a neonatal intensive care unit. These two institutions serve a population of approximately two million people and include the entire population of the state and some regions from neighboring states [13].

In this study, we included all admitted patients that fulfilled the current criteria for SAMM and for NM according to the WHO-Working Group on Maternal Mortality and Morbidity [6]. The first requirement was to be a pregnant woman or to be within 42 days of puerperium, irrespective of the duration and pregnancy condition. Patients were included in the study only once, even if they were admitted on more than one occasion, and were excluded in the case of maternal death.

Three groups were selected according to the definition criteria and defined as: SAMM group, NM group and the control group [6]. This last group was selected from the same hospital, on the same day and with a ratio of two control subjects to one near miss subject. When a case was identified, all patients who were eligible to be controls (admitted on the same day) were listed and numbered and two numbers were selected at random from a covered box. Using such a strategy, bias selection was avoided as each eligible control had the same chance to be selected. The exclusion condition for controls was not having any eligible criteria for SAMM or NM or if they refused to participate.

Every 48 hours, the PI (responsible obstetrician) undertook an active search of cases in both hospitals to identify eligible patients. On the same day, after obtaining written consent from patients, trained medical students conducted the interviews with the patients and reviewed medical records. A pre-coded questionnaire with 59 questions was developed specifically for this study, containing information on sociodemographic profile, prenatal data, clinic and admission conditions, parity, previous pregnancy data, type of delivery, caesarean indications, complications during and after the delivery and perinatal results. Some standardized ratios were calculated, according to the current established definitions by WHO in 2009 [6]. Severe acute maternal morbidity ratio (SAMM-IR) and the overall prevalence were also calculated.

Data was entered in to a computer database using Microsoft Excel 2007. The statistical analysis was performed using SPSS (SPSS Inc., Chicago, IL, USA, version 16.0) for Windows and was double-checked before analysis; *P* <0.05 was considered significant. Categorical variables were expressed as frequency and percentage and were tested using an exact Fisher test for 2x2 tables or a chi-square test for larger tables. For continuous variables, after testing normality with a Shapiro-Wilk test we used a student’s *t* test for the normal and a U-Mann–Whitney for the non-normal variables. An *Odds Ratio* was used when NM and control values differed. Binary logistic regression multivariate analysis was used as a backward method, NM experience as the dependent variable. Independent variables were included when the *p*-value <0.150 and represented at least 80% of the sample in each group. Any variables that did not reach the above conditions were included in the model. The confidence interval (CI) was 95% and a *p*-value of <0.05 was considered statistically significant. The number of valid cases is indicated for each variable and missing data were excluded of from the analysis.

This study was approved by the Ethics Committee in Research of the Federal University of Sergipe, Brazil (Protocol number: 0184.0.107.000-11).

The authors used the STROBE statement and revised the paper accordingly.

## Results

During the study period, there were 16,243 LB deliveries and we identified 1,102 SAMM cases, 77 NM and 17 MD. In order to compare the 77 NM cases, we randomly selected 151 controls. There were 94 women with a severe maternal outcome ratio (SMOR) (77NM + 17MD), with a ratio of 5.8 cases/1,000 LB. The maternal NM incidence ratio was 4.7 cases/1,000 LB and the SAMM incidence ratio was 67.8 cases/1,000 LB. The total prevalence (SAMM + NM) was 72.6 cases/1,000 LB (7.3%). The maternal near miss: mortality ratio was 4.5:1, the mortality index was 18% and the mortality ratio for the studied population was 104.6 cases/100,000 LB [Table 1].

**Table 1:** Indicators of maternal morbidity and mortality for the population in the referral maternity wards of Sergipe

**Coeficients**	**Ratios**
**Women with SMOR**	5.8 cases/1000LB
**NM incidence ratio**	4.7 cases/1000LB
**SAMM incidence ratio**	67.8 cases/1000LB
**Total prevalence (SAMM + NM)**	72.6 cases/1000LB (7.3%)
**Maternal near miss: mortality ratio**	4.5 cases/1000LB
**Mortality index**	18%
**Mortality ratio for the population**	104.6 cases/1000LB

*SMOR*: severe maternal outcome ratio; *LB*: liveborn infants (16,243LB); *NM*: maternal near miss; *SAMM*: severe acute maternal morbidity.

The main conditions diagnosed in SAMM cases were: 67.5% with hypertensive disorders, 61.7% with severe management indicators, 15.4% with hemorrhagic disorders and 8.5% with other systemic disorders. For the NM cases, the main criteria were: 87.1% management-based criteria, 41.4% clinical criteria and 21.4% laboratory-based criteria. The sum was higher than 100% because frequently patients met more than one eligible criterion [Table 2].

**Table 2:** The main diagnosed conditions for SAMM/NM cases in the referral maternity wards in Sergipe

	**SAMM (n = 1102)**		**NM (n = 77)**	
**Criteria**	**n**	**%**	**n**	**%**
**Haemorrhagic disorders**	152	15.4	-	-
**Hypertensive disorders**	666	67.5	-	-
**Other systemic disorders**	84	8.5	-	-
**Severe management indicators**	607	61.7	-	-
**Clinical criteria**	-	-	29	41.4
**Laboratory-based criteria**	-	-	15	21.4
**Management-based criteria**	-	-	61	87.1

The sum totaled more than 100% because the patients had were eligible for more than one SAMM/NM criterion. *NM*: maternal near miss; *SAMM*: severe acute maternal morbidity.

While 56.9% patients meeting SAMM criteria and 53% meeting NM criteria were referred to the two reference hospitals by other health services; 58% of the controls came from their own residences (*p* = 0.001). Conditions diagnosing SAMM/NM were associated with higher age (*p* = 0.018), earlier gestational age at admission (*p* = 0.016), lack of antenatal care (*p* = 0.013), previous abortion and caesarean section (*p* = 0.001 for both), higher rates of current caesarean section (*p* = 0.001), unconscious state on admission (*p* = 0.001), and lower birth weight babies and newborn perinatal period with an unfavorable prognosis (*p* = 0.001 for both). SAMM cases had higher mean for systolic and diastolic blood pressure (*p* = 0.001 for both), [Tables 3 and 4].

**Table 3:** Sociodemographic profile showing the distribution of cases of Severe Acute Maternal Morbidity/Near Miss (SAMM, NM) and controls in the reference maternity wards

**Sociodemographic Variables**	**SAMM (n = 1102) n % or mean ± SD**		**NM (n = 77) n % or mean ± SD**		**Controls (n = 151) n % or mean ± SD**		***p *** **value**
**Provenance**							0.001
Home	400	43.1	31	47	88	58	
Health service	527	56.9	35	53	63	42	
**Age**							0.068
Until 35 years	800	82	51	73.9	131	86.8	
≥ 35 years	176	18	18	26.1	20	13.2	
**Years of education**^¥^							0.414
Until 8 years	523	56.9	37	58.7	76	51.4	
≥8 years	396	43.1	26	41.3	72	48.6	
**Marital status**							0.191
Not married	166	17.8	14	21.5	19	12.6	
Married	768	82.2	51	78.5	132	87.4	
**Work**							0.898
No	643	69.2	45	69.2	101	67.3	
Yes	286	30.8	20	30.8	49	32.7	
**Monthly income**^#^							0.002
Until US$300	532	58.3	36	56.2	92	60.9	
≥ US$300	380	41.7	28	43.8	59	39.1	
Race*							0.934
Not white	777	85.1	54	84.4	130	86.1	
White	136	14.9	10	15.6	21	13.9	
**Head of household**							0.211
Other**	371	40.9	28	43.8	51	33.8	
Husband	539	59.1	36	56.2	100	66.2	
**Health plan**							0.942^§^
Public system	893	95.3	62	95.4	142	94.7	
Health insurance	44	4.7	3	4.6	8	5.3	
**Use of alcohol**							0.714
Yes	184	20.2	15	23.4	28	18.5	
No	729	79.8	49	76.6	123	81.5	
**Smoking**							0.390
No	65	7.1	7	10.9	14	9.3	
Yes	846	92.9	57	89.1	137	90.7	

*AV*: average; *SD*: standard deviation; ^¥^: Normal distribution (KRUSKAL-WALLIS) tested by ANOVA; ^#^values based in a national classification of income (ABEP 2008) [14] *perception of the interviewee; **other: the patient herself or her mother or father. The n value for each variable may differ because some cases and controls were still pregnant or the available data were collected from medical records or the patient did not know the answer. § Fisher exact test. The variables presented in the table were chosen by statistical significance or clinical relevance.

**Table 4:** Clinical and obstetric profile of distribution of cases of Severe Acute Maternal Morbidity/Near Miss (SAMM/NM) and controls in the reference maternity wards

**Clinical obstetric variables**	**SAMM (n = 1102) n % or mean ± SD**		**NM (n = 77) n % or mean ± SD**		**controls (n = 151) n % or mean ± SD**		***p *** **value**
**Gestational age at admission**	32.9 ± 9.9		31.9 ± 9.9		35.2 ± 5.9		0.016
***Status***							^§^0.001
Pregnant	119	12	7	10	36	23.8	
Puerperium	731	74.1	50	71.4	112	74.2	
Postoperative^¥^	137	13.9	13	18.6	3	2	
**B.M.I.**							0.086
≥26,9	291	32.2	16	25.4	36	24	
Until 26,9	614	67.8	47	74.6	114	76	
**Antenatal care**							0.013
No	102	11	11	17.2	7	4.7	
Yes	822	89	53	82.8	143	95.3	
**Previous caesarean**							0.004
Yes	562	58.9	38	56.7	67	44.4	
No	392	41.1	29	43.3	84	55.6	
**Previous abortion**							0.001
Yes	316	33.1	25	37.9	28	18.5	
No	638	66.9	41	62.1	123	81.5	
**Current type of delivery**							0.001
Caesarean	545	72.8	38	74.5	62	55.4	
Vaginal	204	27.2	13	25.5	50	44.6	
**Systolic blood pressure**	147.9 ± 3.9		128.8 ± 27.7		122.5 ± 17.8		0.001
**Diastolic blood pressure**	96.5 ± 21.4		83.0 ± 21.7		78.9 ± 12.7		0.001
**Level of consciousness on admission**							^§^0.001
Not conscious	76	7.9	16	23.5	3	2	
Conscious	882	92.1	52	76.5	148	98	

^¥^: postoperative of laparotomy or curettage after abortion or delivery. The n value for each variable may differ because some cases and controls were still pregnant or the available data were collected by information from medical records or the patient did not know the answer. Other variables were tested, but they were not statistically significant. § Fisher exact test. The variables presented in the table were chosen by statistical significance or clinical relevance.

When NM cases and controls were compared, a previous history of abortion [OR = 2.7 (CI = 1.4-5.4)] and caesarean section [OR = 1.9 (CI = 1.0-3.6)], higher rates of caesarean section [OR = 2.4 (CI = 1.1-4.9)] and being unconscious on admission more often [OR = 15.2 (CI = 4.3-54.2)] were notable differences between the two groups [Table 5]. Perinatal unfavorable prognosis, through the destination of the LB (ICU/death vs. with the mother) [OR = 8.1 (CI = 3.7-17.7)] was statistically significant.

**Table 5:** Comparison between NM cases and controls in the reference maternity wards of Sergipe (2011/2012)

**Variables**	**NM n % or mean ± SD**		**Controls n % or mean ± SD**		***p *** **value**	***Odds ratio***	***p *** **value multivariate**
***Status***							
Pregnant	7	10	36	23.8	0.016	2.82 (1.19-6.70)	0.022
Puerperium/postoperative^¥^	63	90	115	76.2			
**Marital *status***							
Not married	14	21.5	19	12.6	0.093	1.90 (0.89- 4.09)	0.125
Married	51	78.5	132	87.4			
**Years of education**							
Until 8 years	37	58.7	76	51.4	0.325	1.35 (0.74-2.45)	
≥ 8 years	26	41.3	72	48.6			
**Age**							
Until 35 years	51	73.9	131	86.8	0.019	2.31 (1.13-4.72)	0.140
≥ 35 years	18	26.1	20	13.2			
**Previous abortion**							
Yes	25	37.9	28	18.5	0.002	2.68 (1.41-5.10)	<0.001
No	41	62.1	123	81.5			
**Personal history of risk**							
Yes	22	34.4	33	22	0.058	1.86 (0.98-3.54)	0.171
No	42	65.6	117	78			
**Conducting prenatal**							
No	11	17.2	7	4.7	0.003*	4.24 (1.56-11.51)	0.080
Yes	53	82.8	143	95.3			
**Gestational antecedent (≥G2)**^**§**^							
Caesarean	47	71.2	85	56.3	0.038	1.92 (1.03-3.58)	
Vaginal	19	28.8	66	43.7			
**Current type of delivery**							
Caesarean	38	74.5	62	55.4	0.020	2.36 (1.13-4.90)	
Vaginal	13	25.5	50	44.6			
**Previous caesarean section**							
Yes	38	56.7	67	55.6	0.092	1.64 (1.41-5.40)	0.039
No	29	43.3	84	44.4			
**Level of counsciousness on admission**							
Not conscious	16	23.5	3	2	<0.001*	15.18 (4.25-54.21)	<0.001
Conscious	52	76.5	148	98			

^¥^: postoperative of laparotomy or curettage after abortion or delivery; The n value for each variable may differ because some cases and controls were still pregnant or the avaiable data were collected by information from medical records or the patient did not know the answer. *Fisher’s exact test. The variables presented in the table were chosen by statistical significance or clinical relevance. ^§^Just for patients in the second gestation or more.

In regard to the number of the eligible criteria per case, the univariate analysis demonstrated that 74.5% were eligible for more than one SAMM or NM criterion. During the study period there were 17 MD and the main causes were: hemorrhage (41.2%), hypertensive disorders (17.5%), embolism (11.8%), abortion (11.8%), baseline diseases complicated by pregnancy (11.8%) and pelvic infection (5.9%).

The variables that satisfied the conditions to be included in the multivariate model were: patient’s *status,* age, marital *status*, previous cesarean section, previous abortion, antenatal care, personal history of risk and level of consciousness. After the variables were evaluated together, four of the eight remained statistically significant: patient’s *status* (*p* = 0.020), previous cesarean section (p = 0.039), previous abortion (p < 0.001) and level of consciousness (p < 0.001) [Table 5].

## Discussion

Using the official WHO definitions, we could identify the situations characterized as SAMM and NM that occurred in patients in Sergipe state. The main condition diagnosing SAMM was hypertensive disorders. For NM, 87.1% were more frequently associated with management-based criteria and secondly with clinical criteria (21.4%). These numbers reinforce the specificity of the management problems -based criteria for NM in detecting patients with real severe obstetric [15]. Hypertension was the most frequent obstetric pathology and was the main cause of morbidity in the SAMM group. This was similar to the results reported for the rest of the country and to results from other developing countries [1,16].

However, inside the group of management-based criteria (the most common for NM) hemorrhage was the most frequent, highlighting the importance of this condition in the context of NM and the correlation with death. The maternal mortality ratio for the studied population was approximately 57% higher than the Brazilian national average (104.6/100,000 LB vs. 60.1/100,000 LB). The proportion of maternal near miss to maternal deaths confirms other reports in the literature, which state that the condition is at least four times more frequent, which justify the study of NM [17]. The audit of these cases facilitates our understanding of the real demands of each health service, enabling us to improve the quality of obstetric care and to reduce the incidence of these preventable causes of death [6].

In terms of the sociodemographic variables that may be associated with SAMM and NM, the correlation between these events and higher age of the mother could be due to the fact that older women acquire chronic diseases throughout life. It may influence the gestational prognosis, increasing the chance of complications. Some studies suggest an association between SAMM/NM and marital status and education [15]. This was not observed in this study, possibly because this particular population is highly homogeneous: people live under similar socioeconomic conditions, they are predominantly not white, they are economically inactive and are dependent on public health services.

In addition, there was a significant association between SAMM/NM and patients who had a history of previous abortion, confirming findings from the literature [18]. Previous caesarean and current caesarean section were also statistically significant, but this occurred frequently even among the controls. In Brazil, the caesarean rate is considered a public health problem and this kind of delivery by itself increases the chances of repetition in the future [19,20].

Caesarean section, combined with an earlier gestational age on admission of the SAMM and NM cases, resulted in adverse perinatal outcomes and consequently more admissions to the intensive care unit and an increase in neonatal mortality ratios. In addition, an absence of prenatal attendance, and therefore lack of adequate monitoring, compounds a potentially life threatening condition by delaying an essential diagnosis and treatments that result in a good outcome for the pregnancy.

The univariate analysis indicated that the NM group presented with, in the majority of the cases (74.1%), more than one eligible criterion when compared to the SAMM group (25.6%). This shows how complex the management of NM cases can be, added to the severity of the criteria itself, suggesting a syndromic characteristic of NM and confirming the biological *continuum* theory where the lack of intervention may culminate in a real risk of death [7].

The results of the multivariate analysis indicate that a patient’s previous obstetric history is valuable. Knowledge of a previous caesarian section is important because this kind of delivery is only justified in case of risk, which may reoccur during the patient’s next pregnancy, despite the high rates of unjustified caesarean section in Brazil. Little is known about the associated risk between previous abortion and SAMM/NM (particularly hemorrhagic and infectious complications) [21]. However, this may be an important risk factor that is not commonly reported because some women tend to omit information on the practice of abortion, especially in countries where the legislation is restrictive [22,23]. Patients in the puerperium or that were submitted to curettage or postoperative had more SAMM/NM events because the event delivery/surgery itself may be the reason for the complication. The level of consciousness was associated with SAMM and NM because some patients exhibited high levels of severity since their hospital’s admission.

There were a number of limitations to this study, such as the lack of electronic medical record storage making it difficult to have access to cases, limitations to the diagnostic and therapeutic resources in the services and the overcrowding of the maternity hospitals. We have exhaustively revised patients’ records and made use of the available hospital database to minimize information loss. The lack of antenatal care, the lack of resources (e.g., no maternal intensive care unit available in the high risk maternity) were the main problems which led us to classify some patients as SAMM or NM, but the same criteria were used throughout the entire study without investigator interference.

SAMM and NM situations were prevalent in the studied population and some risk factors seem to be associated with the event, particularly previous gestational antecedents. The use of the WHO maternal near miss criteria was feasible in the Northeast of Brazil and provided useful information. Decreasing maternal mortality is more than ever a matter relevant to human rights and everyone has a part to play: government, human rights organizations, health service providers and civil society [24]. From an epidemiological point of view, it is necessary to enhance the coverage and the quality of antenatal care, to improve the infrastructure of maternity wards to enable proper management of severe complications and to promote the work of multidisciplinary obstetric teams. Protocols based on adverse situations like NM, which identifies the exact point of failure prior to death, may allow us to recommend interventions that save lives.

## Conclusions

The prevalence of SAMM and NM in the state of Sergipe was high, though within the ranges described in the literature. The mortality ratio for the population studied was also high, at least 40% higher than the Brazilian average. Hypertensive disorders were more commonly associated with morbidity, while hemorrhage was the main cause of mortality in this population. The only significant variable from the sociodemographic profile was higher age. Patients who have previously undergone a caesarean and/or an abortion should be noted and these should be considered obstetrical antecedents. The lack of regular antenatal care, high rates of caesareans and prematurity with adverse perinatal outcomes were also statistically significant. The existence of more than one criterion for NM eligibility demonstrates the complex management required for these patients and highlights the biological *continuum* for this situation.

## Abbreviations

CI: Confidence interval; LB: Live birth; MD: Maternal death; NM: Near miss; SAMM: Severe acute maternal near miss; SAMM IR: Severe acute maternal near miss incidence ratio.

## Competing interests

The authors declare that they have no competing interests.

## Authors’ contributions

LPG and RQG designed the study and participated in all phases of the study. FFM, CMM, RFR and KNG collected data and LPG supervised it. FAP performed the statistical analysis. All authors read, reviewed and agreed with the final version.

## Authors’ information

^1^Master in Health Sciences,^2^ Adjunct Professor and Dean of Dentistry, Federal University of Sergipe, ^3,4,5,6^ Medical students, Federal University of Sergipe, ^7^ Associate Professor of Pediatrics – Graduate Nucleus of Medicine, Federal University of Sergipe.

## Pre-publication history

The pre-publication history for this paper can be accessed here:

http://www.biomedcentral.com/1471-2393/14/25/prepub

## References

[B1] Trends in maternal mortality: 1990 to 2010 2012 Geneva: World Health Organization http://whqlibdoc.who.int/publications/2012/9789241503631_eng.pdf

[B2] Filippi V Ronsmans C Campbell O Graham WJ Mills A Borghi J Maternal health in poor countries: the broader context and a call for action Lancet 2006 368 1535 1541 10.1016/S0140-6736(06)69384-7 17071287

[B3] Lawn JE Cousens S Zupan J Neonatal Survival Steering Team:4 million neonatal deaths: When? Where? Why? Lancet 2005 365 9462 891 900 10.1016/S0140-6736(05)71048-5 15752534

[B4] DATASUS Maternal Deaths 2010 http://www.datasus.gov.br

[B5] Abouzahr C Wardlaw T Maternal mortality at the end of the decade: signs of progress? Bull World Health Organ 2001 79 561 573 11436479 PMC2566451

[B6] Say L Souza JP Pattinson RC Maternal near miss – towards a standard tool for monitoring quality of maternal health care Best Pract Res Clin Obstet Gynaecol 2009 23 287 296 10.1016/j.bpobgyn.2009.01.007 19303368

[B7] Geller SE Rosenberg D Cox SM Killpatrick S Defining a conceptual framework for near miss maternal morbidity Am J Med Women Assoc 2002 57 135 139 12146602

[B8] Stones W Lim W Al-Azzawi F Kelly M An investigation of maternal morbidity with identification of life – threatening near miss episodes Health Trends 1991 23 13 15 10113878

[B9] Penny G Brace V Near miss audit in obstetrics Curr Opin Obstet Gynecol 2007 19 2 145 150 10.1097/GCO.0b013e328014a860 17353683

[B10] Tulçalp O Hindin MJ Souza JP Chou D Say L The prevalence of maternal near miss: a systematic review BJOG 2012 119 6 653 661 10.1111/j.1471-0528.2012.03294.x 22489760

[B11] Souza JP Cecatti JG Parpinelli MA Souza MH Serruya SJ Systematic review of maternal near miss Cad Saude Publica 2006 22 2 255 264 10.1590/S0102-311X2006000200003 16501738

[B12] Souza JP Cecatti JG Haddad SM Parpinelli MA Costa ML Katz L Say L The WHO Maternal Near-Miss Approach and the Maternal Severity Index Model (MSI): Tools for Acessing the Management of Severe Maternal Morbidity PLoS One 2012 7 HTTP://www.plosone.org 10.1371/journal.pone.0044129 PMC3430678 22952897

[B13] IBGE census 2010 HTTP://www.ibge.com.br

[B14] ABEP Associação Brasileira de Empresas de Pesquisa. Critério de classificação economica Brasil 2008 http://www.abep.org

[B15] Geller SE Rosenberg D Cox SM Brown M Simonson L Kilpatrick SA A scoring system identified near miss maternal morbidity during pregnancy J Clin Epidemiol 2004 57 7 716 720 10.1016/j.jclinepi.2004.01.003 15358399

[B16] Souza JP Cecatti JG Parpinelli MA Factors associated with the severity of maternal morbidity for the characterization of near miss Rev Bras Ginecol Obstet 2005 27 4 197 203 10.1590/S0100-72032005000400006

[B17] Pattinson RC Hall M Near misses: a useful adjunt to maternal death enquires Br Med Bull 2003 67 231 243 10.1093/bmb/ldg007 14711767

[B18] Camargo RS Santana DS Cecatti JG Pacagnella RC Tedesco RP Mello JR Elias F Sousa MH Severe maternal morbidity and factors associated with the occurrence of abortion in Brazil Int J Gynecol Obstet 2011 112 88 92 10.1016/j.ijgo.2010.08.013 21130447

[B19] Proportion of caesarean section 2010 RIPSA [http://tabnet.gov.br/cgi/tabcgi.exe?idb2011/f08.def. Acessado em 13/12/2012]

[B20] Cecatti JG Pires HM Faúndes A Duarte Osis MJ Associated factors with vaginal delivery after caesarean section in brazilian women Rev Panam Salud Publica 2005 18 2 107 113 10.1590/S1020-49892005000700005 16156961

[B21] Singh S Hospital admissions resulting from unsafe abortion: estimates from 13 developing countries Lancet 2006 368 9550 1887 1892 10.1016/S0140-6736(06)69778-X 17126721

[B22] Osis MJD Hardy E Faundes A Rodrigues T Dificulties encountered in gathering information on illegal abortion in the population of women Rev de Saúde Pública 1996 30 5 444 451 10.1590/s0034-89101996000500007 9269094

[B23] Sedgh G Henshaw SK Bankole A Dresher J Induced abortion: estimated rates and trends worldwide Lancet 2007 370 9595 1338 1345 10.1016/S0140-6736(07)61575-X 17933648

[B24] Pillay N Maternal mortality and morbidity: a human rights imperative Lancet 2013 381 1159 1160 10.1016/S0140-6736(13)60135-X 23561986

